# Hydroxyapatite Film Coating by Er:YAG Pulsed Laser Deposition Method for the Repair of Enamel Defects

**DOI:** 10.3390/ma14237475

**Published:** 2021-12-06

**Authors:** Liji Chen, Shigeki Hontsu, Satoshi Komasa, Ei Yamamoto, Yoshiya Hashimoto, Naoyuki Matsumoto

**Affiliations:** 1 Department of Orthodontics, Osaka Dental University, 1-5-17 Otemae, Chuo-ku, Osaka 540-0008, Japan; chen-li@cc.osaka-dent.ac.jp (L.C.); naoyuki@cc.osaka-dent.ac.jp (N.M.); 2 Department of Biomedical Engineering, Faculty of Biology-Oriented Science and Technology, Kindai University, 930 Nishimitani, Kinokawa 649-6493, Japan; hontsu@waka.kindai.ac.jp (S.H.); ei@waka.kindai.ac.jp (E.Y.); 3 Department of Removable Prosthodontics and Occlusion, Osaka Dental University, 1-5-17 Otemae, Chuo-ku, Osaka 540-0008, Japan; komasa-s@cc.osaka-dent.ac.jp; 4 Department of Biomaterials, Osaka Dental University, 8-1 Kuzuha Hanazono-cho, Hirakata 573-1121, Japan

**Keywords:** hydroxyapatite coating, Er:YAG laser, pulsed laser deposition, enamel defects

## Abstract

There are treatments available for enamel demineralization or acid erosion, but they have limitations. We aimed to manufacture a device that could directly form a hydroxyapatite (HAp) film coating on the enamel with a chairside erbium-doped yttrium aluminum garnet (Er:YAG) laser using the pulsed laser deposition (PLD) method for repairing enamel defects. We used decalcified bovine enamel specimens and compacted α-tricalcium phosphate (α-TCP) as targets of Er:YAG-PLD. With irradiation, an α-TCP coating layer was immediately deposited on the specimen surface. The morphological, mechanical, and chemical characteristics of the coatings were evaluated using scanning electron microscopy (SEM), scanning probe microscopy (SPM), X-ray diffractometry (XRD), and a micro-Vickers hardness tester. Wear resistance, cell attachment of the HAp coatings, and temperature changes during the Er:YAG-PLD procedure were also observed. SEM demonstrated that the α-TCP powder turned into microparticles by irradiation. XRD peaks revealed that the coatings were almost hydrolyzed into HAp within 2 days. Micro-Vickers hardness indicated that the hardness lost by decalcification was almost recovered by the coatings. The results suggest that the Er:YAG-PLD technique is useful for repairing enamel defects and has great potential for future clinical applications.

## 1. Introduction

The high bond strength between the tooth enamel and bracket in orthodontic treatment results in enamel damage on bracket removal. Upon debonding, the bracket fails at the bracket–adhesive interface, leaving behind all or most of the adhesive on the enamel, requiring removal by rotary burs at a low speed [1]. The remanent adhesive on the surface of the teeth is not ideal for strength and luster. Furthermore, the enamel rods that were decalcified during etching are abraded, resulting in thinner enamel. The enamel on the tooth surface is acellular and scarcely self-repaired after damage after the time of tooth eruption [2,3]. As the adhesive used for orthodontic treatment is composite resin, marginal gaps and microleakage between fillings and teeth are undesirable [4]. Decalcification of the enamel is a common side effect of orthodontic treatment with fixed appliances; however, strategies to prevent it are limited.

Hydroxyapatite (HAp, Ca_10_(PO_4_)_6_ (OH)_2_) is used in a variety of applications, such as medical and dental devices for implantation into living bodies [5]. Therefore, nonstoichiometric fluoridated carbonate HAp crystal, the primary mineral phase (≈96 weight %) of enamel, is the ideal material to treat the enamel surface post-orthodontic decalcification [6]. In general, it is ideal to restore and preserve dentin using hydroxyapatite, which is the main constituent of teeth. However, adhesion between ceramics is extremely difficult to achieve owing to the difference in roughness.

The thin layer coating of HAp is one of the most useful treatments for enamel surfaces. It has been reported that an HAp film can be created via powder jet deposition, by manipulating the blasting nozzle above human enamel for restoration [7]. Alternatively, our team created an ultra-thin apatite sheet attachment method, that is, an HAp sheet with a thickness of 1–10 μm with flexibility on the tooth surface and integration with tooth structure [8]. In our previous study, the HAp bulk target was irradiated with pulsed laser deposition (PLD) using an excimer laser [9], and the bonds between the constituent target atoms were broken using light energy to cause PLD. We achieved a successful application of a thin HAp coating with a thickness of 1 μm or less on the titanium implant base material, with the constituent HAp atoms scattered from the target surface. However, neither of these methods has been applied to clinical practice thus far.

Erbium-doped yttrium aluminum garnet (Er:YAG) lasers are widely used in operative dentistry for the treatment of hard and soft tissue diseases of the oral cavity and in endodontics [10,11]. In the present study, the wavelength of the Er:YAG laser used in the PLD method is as long as 2.94 μm, so the photon energy is low, about 0.42 eV. Since the energy of photons cannot directly break the bonds between atoms, its function of ablation causes instantaneous evaporation of the hydration shell of laser targets, thus increasing pressure, producing vapor within the laser targets, and provoking “micro-explosions” that cause the mechanical breakdown of the laser targets and physically contribute to the ablation process [12].

Based on the above principle, in the present study, we propose a strategy to treat the enamel by directly depositing coatings of α-tricalcium phosphate (α-TCP), the precursor of Hap, on demineralized bovine enamel surfaces under atmospheric pressure and at room temperature. We speculated that the constituent atoms of α-TCP scattered from the α-TCP target by irradiation with Er:YAG laser light could deposit on the dental tissue, and also the increase in the particle surface area expedited the hydrolysis of the α-TCP into HAp.

This paper reports a treatment of defected enamel by directly depositing α-TCP coatings on demineralized bovine enamel surfaces under the specific Er:YAG-PLD conditions based on the clinical environment. After the hydrolytic differentiation process, we investigated whether the hierarchical structure and the mechanical properties of an HAp film coating will be identical to those of the natural enamel.

## 2. Materials and Methods

### 2.1. Preparation of Targets for Er:YAG-PLD

**Figure 1:**
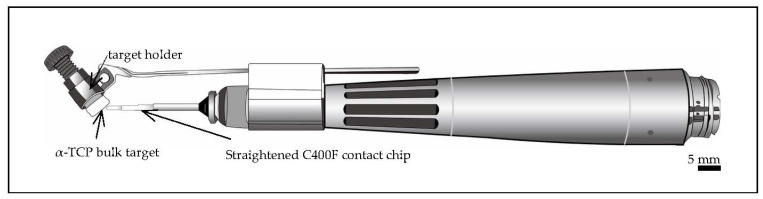
The schematic graph of handpiece consisting of a straightened C400F contact tip and α-TCP bulk target.

The α-TCP bulk (precursor of HAp) was used as the target material for Er:YAG-PLD. Raw α-TCP powder (α-TCP-B, Taihei Chemical Industrial, Osaka, Japan) was compressed using a hydraulic press under a pressure of 0.3 MPa for 60 s to form compacted targets, which were 5 mm in diameter and about 7 mm in length (Figure 1). The targets were soaked in pure water for ablation during Er:YAG-PLD.

### 2.2. Preparation of Bovine Tooth Slabs (Specimens)

Freshly extracted rectangular bovine teeth (5.0 × 7.0 × 2.0 mm^3^) were polished using a silicon carbide abrasive paper until #4000. The discs were etched with 35% phosphoric acid (Opal) for 30 s and then cleaned in deionized water using an ultrasonic bath for 10 min. The specimens (a) were prepared at room temperature.

### 2.3. Chairside Er:YAG-PLD Device

An Er:YAG laser unit (Erwin AdvErl Unit; Morita Manufacturing, Kyoto, Japan) was used for the PLD. The unit’s specifications were as follows: wavelength, 2.94 µm; pulse oscillation energy output, 30–350 mJ/pulse, and repeat pulse frequency settings, 1, 3, 5, 10, 20, and 25 pps. The unit consisted of an optical fiber transmission cable, and the laser beam was delivered through the contact tip. We used a custom-made handpiece with a target holder and a straightened C400F contact tip (Figure 1) (Morita Manufacturing, retrofit) for Er:YAG-PLD. The included angle of the target holder and the tip was set at 30–60°. After filling the target holder with soaked α-TCP bulk, laser irradiation was conducted at 300 mJ/pulse, 5 pps. The laser tip was positioned at 1 mm [13] from the target, and the whole unit was positioned less than 1 mm from the specimens. We manually moved the Er:YAG-PLD handpiece horizontally to obtain a megascopic α-TCP coating.

### 2.4. Preparation of Artificial Saliva

The bovine tooth disks were soaked in artificial saliva (1118.1 mg KCl, 340.4 mg KH_2_PO_4_, 288 mg N_2_HPO_4_, 496.5 mg NaCl, 30.5 mg MgCl_2_·6H_2_O, 55.3 mg CaCl_2_, and 126.1 mg NaHCO_3_ in 1000 mL deionized water; pH = 6.8) [14,15,16] at a temperature of 37 °C for 2 days immediately after Er:YAG-PLD.

### 2.5. Microstructural Analysis

After drying, the samples were sputter-coated with ≈5 nm osmium and were examined using a scanning electron microscope (SEM) (S-4800; Hitachi, Tokyo, Japan). Initially, the specimens were analyzed in a panoramic view, and photomicrographs of the most representative areas of each group were obtained at a standardized magnification. For the cross-section SEM images, the specimens with HAp coating were embedded in crystal resin and cut from the center.

### 2.6. Elemental Analysis

The determination of the contents of elements was performed using energy-dispersive X-ray spectroscopy (EDS) (JED-2300, JEOL) on the surface of bovine teeth and HAp film coatings.

### 2.7. Toothbrush Abrasion and Its Measurements

Brushing tests were conducted to evaluate the adhesion between the HAp film coating and enamel surface. During the brushing tests, a vertical force of 150 g [17,18] was applied to a toothbrush (#211, Sunstar Co., Osaka, Japan) [19] which moved horizontally at 90 strokes/min for 2 min in the air at room temperature. The specimens were carefully rinsed with deionized water after brushing and dried for 5 s with oil-free air. The remaining HAp film coating was confirmed by SEM. We measured the roughness of the bovine enamel and deposited coating before and after the brushing tests using a scanning probe microscope (SPM; SPM-9700, Shimadzu Co., Kyoto, Japan). The brushing tests were repeated on two polished bovine enamel slabs and two decalcified specimens with HAp film coatings. Five indentations were made for each specimen, and the results were recorded. The roughness data were statistically compared by one-way ANOVA and Tukey–Kramer test.

### 2.8. Mechanical Property Measurements

The microhardness of the specimens was measured with a micro-Vickers hardness tester [20] using a 200 g force load and a dwelling time of 20 s. The tests were repeated on six different specimens at the same temperature; five indentations were made for each specimen, and the results were recorded [21].

### 2.9. Crystal Structural Analysis

The samples were analyzed using X-ray diffractometry (XRD) (XRD-6000, Shimadzu Co., Tokyo, Japan) at 20 angles ranging from 2° to 80° with a scan step of 0.02°.

### 2.10. Measurement of Temperature Change during Er:YAG-PLD Procedure

Bovine tooth slabs were placed on a hotplate (37 °C) for 5 min to simulate the intraoral temperature. The temperature change was measured using thermocouples (AS ONE Corporation) located on the surface of specimens during the procedure.

### 2.11. Effect of HAp Film Coating on Cell Attachment

**Figure 2:**
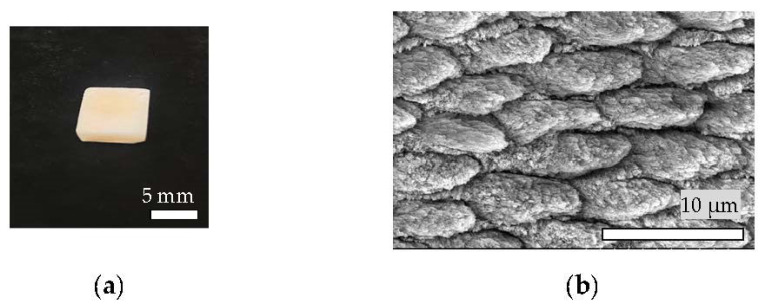
Gross appearance (**a**) and SEM images (**b**) of specimen (10 kV); the interspace of the decalcified bovine enamel prisms can be observed.

A cell attachment study was conducted using human periodontal ligament fibroblasts (HPdLF) [22] (Lonza, Walkersville, Inc., Tokyo, Japan). The culture medium used in all cell cultures was 10% fetal bovine serum in Dulbecco’s modified Eagle’s medium, along with 1% antibiotic/antimycotic (Nacalai Tesque). The cells were cultured at 37 °C in a 5% CO_2_ incubator. The culture medium was exchanged three times per week throughout the study [23]. HPdLF cells were cultured in tissue culture flasks (vent cap) for one week. When they were 80% confluent and contained many mitotic figures throughout the flask, the cells were sub-cultured onto the HAp film coating in 48-well culture plates (Figure 2) at a density of 1000 cells per sample as a cell suspension and incubated in a 5% CO_2_ humidified incubator at 37 °C for 6 h. Subsequently, the culture medium (0.2 mL) was added, and the cells were incubated for another 18 h.

The HAp film coating on the etched bovine enamel was sterilized by autoclaving (121 °C, 125 kPa, 60 min) and furbished [24] (silicon carbide abrasive paper until #4000) for cell culture [25]. For SEM analysis, the HPdLF cells were processed for fixation and then gently rinsed three times with phosphate-buffered saline (PBS). Then, the cells were incubated for 60 min with 4% paraformaldehyde phosphate buffer solution at 4 °C and then rinsed with PBS three times. The cells were dehydrated by increasing the concentrations of ethanol (50, 60, 70, 80, 90, 99, and 99.5%, each for 10 min) and then 3-methylbutyl acetate for 60 min. Then, the liquid inside the cells was exchanged for liquid carbon dioxide (CO_2_) followed by drying in supercritical conditions (CO_2_ critical point temperature and pressure: 31.0 °C and 72.8 kg/cm^2^, respectively). After drying, the samples were sputter-coated with ≈5 nm osmium and observed by SEM [26,27].

### 2.12. Statistical Analysis

A statistical analysis was performed using Statcel 4 software (OMS Publishing Inc., Saitama, Japan). Analysis of the hardness data was conducted using one-way analysis of variance (ANOVA) with Tukey’s post hoc test. The significance level was set to α = 0.05. Additionally, to check the changes in the roughness values of the bovine enamel and HAp film coating before and after the brushing test, an individual t-test was performed.

## 3. Results

### 3.1. Materials Fabrication

**Figure 3:**
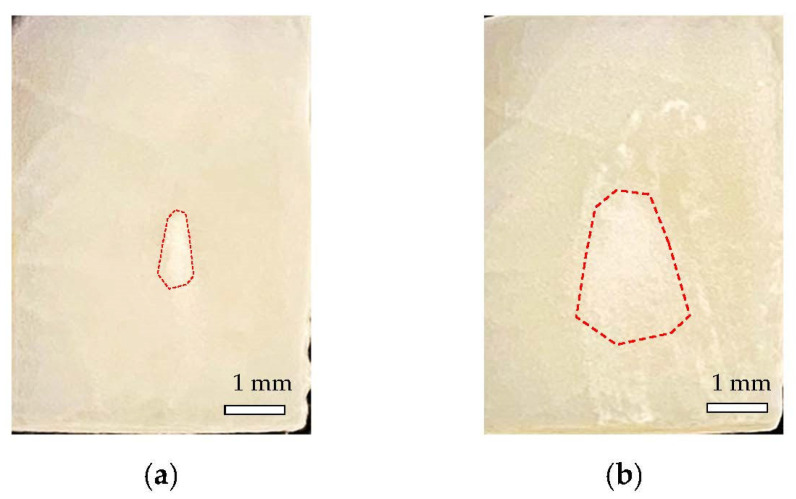
Gross appearances of specimens and the deposited α-TCP layer immediately after the Er:YAG-PLD for (**a**) 1 pulse and (**b**) 25 pulses.

The gross appearances of the α-TCP layer deposited immediately after Er:YAG-PLD for (a) 1 pulse and (b) 25 pulses on specimens are shown in Figure 3. The region inside the red dotted line shows the deposited α-TCP. The α-TCP layer appears to be an opaque white section, and once coated on etched bovine enamel slabs, it will not be removed by air flow or a 10 min ultrasonic bath.

### 3.2. Scanning Electron Microscope Analysis

**Figure 4:**
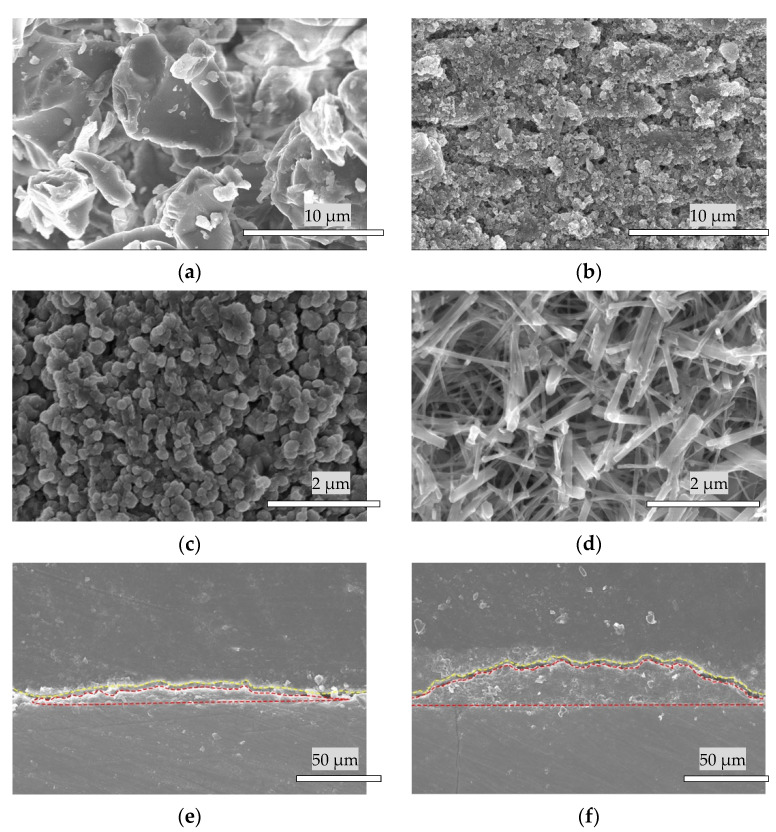
SEM images of (**a**) (10 kV) α-TCP powder, the deposition of α-TCP layer by (**b**) (10 kV) 1 pulse and (**c**) (5 kV) 25 pulses of Er:YAG-PLD. (**d**) (15 kV) Plate-like crystallites of HAp after hydrolyzation by 25 pulses of PLD. The cross-sectional SEM of HAp coated specimens by one pulse (**e**) (10 kV) and 25 pulses of Er:YAG-PLD (**f**) (10 kV) following hydrolyzation.

The most significant images obtained for each group are shown as follows:

Figure 4a shows an image of the raw α-TCP powder (α-TCP-B, Taihei Chemical Industrial, Osaka, Japan) particles. Figure 4b shows the bovine enamel plugged by α-TCP microparticles (majority at a size of 0.2 μm) after one pulse of Er:YAG-PLD. Figure 4c shows an image of the α-TCP microparticle layer after 25 pulses of Er:YAG-PLD, which was followed by cleaning with deionized water in an ultrasonic bath for 10 min and drying by air flow for 15 s. Figure 4d shows that the α-TCP microparticles turned into plate-like crystallites of HAp after hydrolyzation for 48 h. Figure 4e,f are the cross-sectional SEM of HAp coated specimens by one pulse (e) (10 kV) and 25 pulses of Er:YAG-PLD (f) (10 kV) following hydrolyzation. The region above the yellow dotted line is the crystal resin, and the region inside the red dotted line is the deposited Hap film coating.

### 3.3. Surface Microstructure of the Bovine Enamel and HAp Film Coating Deposited Specimens before and after the Brushing Test

**Figure 5:**
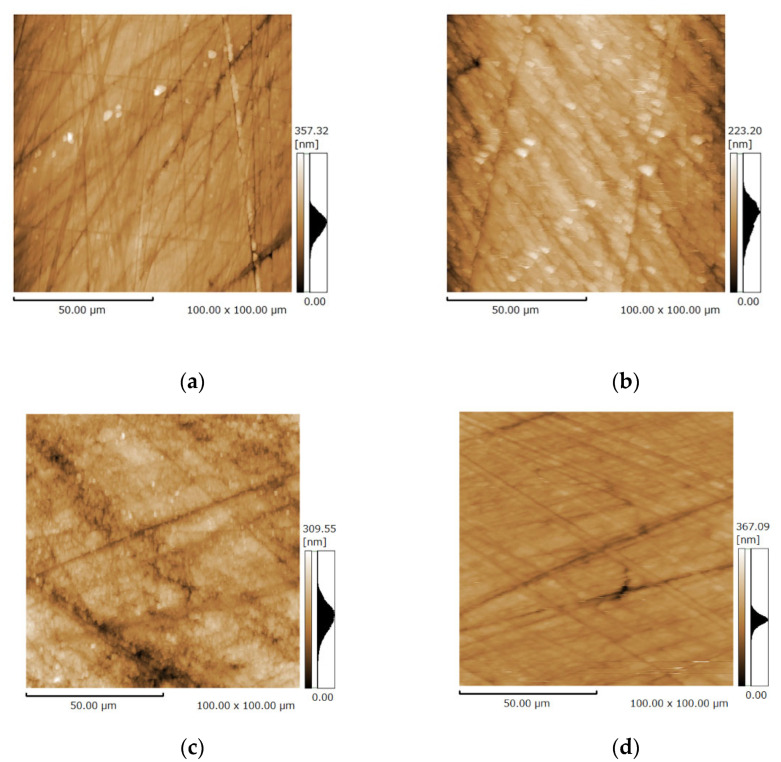
Scanning probe microscopy micrograph of the specimen: (**a**) polished bovine enamel; (**b**) polished hydroxyapatite coating on the specimen; (**c**) bovine enamel after brushing test; (**d**) hydroxyapatite coating after brushing test.

**Figure 6:**
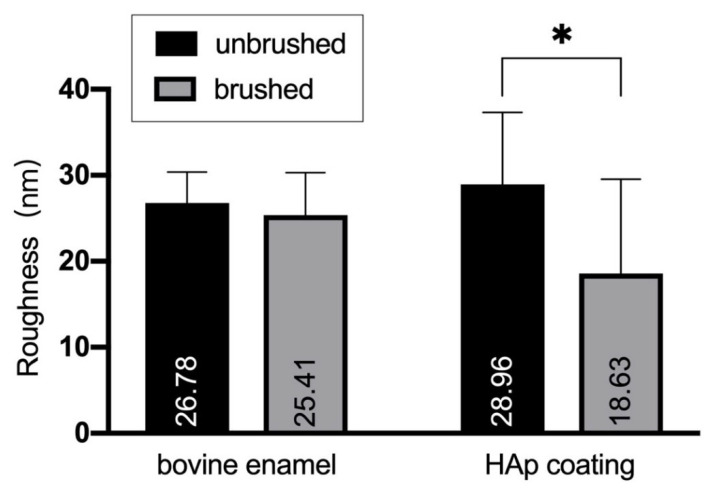
Roughness values of the specimen with and without hydroxyapatite coating before and after the brushing test (*n* = 10). * *p* < 0.05.

The roughness of bovine enamel with and without HAp film coating before and after the brushing test were measured using SPM (Figure 5 and Figure 6).

Figure 6 shows that the roughness values of the bovine enamel and HAp film coating both decreased after the brushing test. After brushing, the roughness of the HAp film coating was significantly reduced (*p* < 0.05), which indicated that the HAp film coating became smoother after tooth brushing.

### 3.4. Hardness Analysis

**Figure 7:**
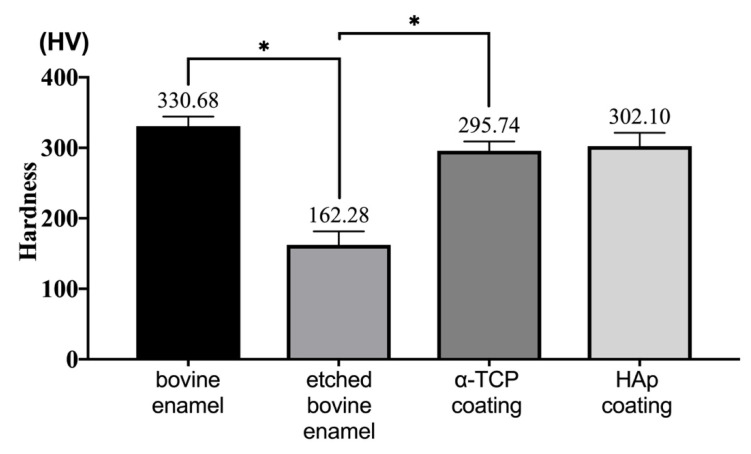
Comparison of the hardness of the bovine dental enamel, etched bovine enamel (specimens), specimens with α-TCP sheet, and specimens with hydroxyapatite coating (*n* = 30). * *p* < 0.05.

Figure 7 shows that the hardness of bovine enamel reduced significantly (from 329 to 164 HV) after etching with 35% phosphoric acid (*p* < 0.05). When the α-TCP coating was formed on the specimen, the hardness significantly increased, and 89.4% of the hardness was recovered from the initial enamel (*p* < 0.05). A hardness of 91.8% was regained when the coatings were converted into HAp. There was no significant difference between the hardness of the α-TCP and HAp film coatings (*p* > 0.05).

### 3.5. Phase Determination by XRD

**Figure 8:**
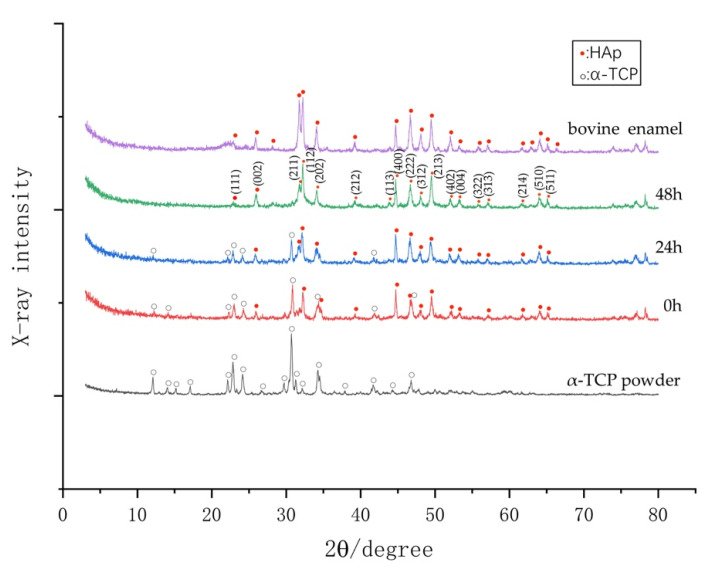
X-ray diffraction patterns of the α-TCP powder, deposited film coating immediately after PLD, coatings after hydrolyzation for 24 h and 48 h, and bovine dental enamel.

Figure 8 shows the X-ray diffraction (XRD) patterns of raw α-TCP powder coating after the Er:YAG-PLD and after being hydrolyzed for 24 and 48 h at 37 °C. Many diffraction peaks matching those of α-TCP were observed in the XRD pattern of the coating that was hydrolyzed for 24 h. With the duration of hydrolyzation, the α-TCP peaks in the XRD pattern tended to decrease, while HAp peaks tended to increase, indicating that the α-TCP coating gradually changed into a crystallized HAp film coating within 48 h. We determined the condition of the remaining HAp film coating on the specimens by SEM. However, the α-TCP peaks were observed even after hydrolyzation for 48 h, and plate-like crystallites of HAp were observed by SEM. This result reveals that α-TCP and HAp coexist in the deposited coating.

### 3.6. Temperature Changes Following Er:YAG-PLD

**Figure 9:**
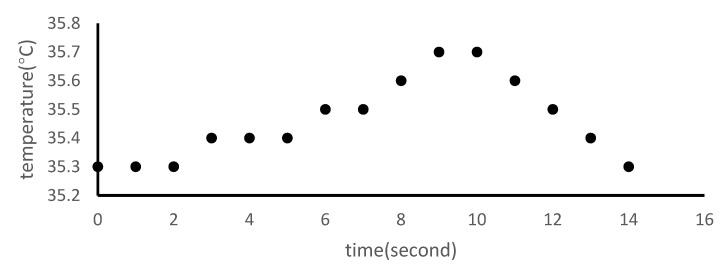
Temperature change during the Er:YAG-PLD procedure.

The temperature line chart (Figure 9) shows that the temperature of the specimens increased by 0.4 °C in 10 s during the Er:YAG-PLD procedure and returned to the initial temperature in 4 s after the Er:YAG-PLD process was over.

### 3.7. Calcium-to-Phosphate (Ca/P) Analysis of HAp Film Coating by Energy-Dispersive X-ray Spectroscopy (EDS)

According to energy-dispersive spectrometry, compositional analysis of the HAp film coating on the bovine enamel and specimens with HAp film coating (Table 1) revealed peaks of sodium and magnesium other than calcium, phosphate, carbon, and oxygen on the HAp film coating. EDS analyses of bovine teeth led to an average Ca/P ratio of 1.31 ± 0.02, and average Ca/P ratio of HAp film coating on specimen was 1.45 ± 0.05 (Table 2).

### 3.8. Cell Attachment of HAp Film Coating

**Figure 10:**
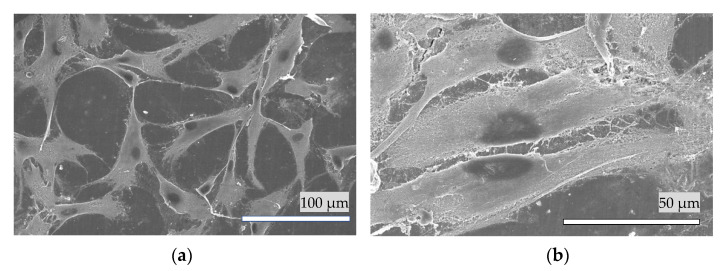
A typical SEM image of the HPdLF cells attached on the surface of (**a**) the hydroxyapatite coating and (**b**) bovine enamel.

Figure 10a,b shows the living HPdLF cells attached to the HAp film coating (a) and bovine enamel (b). The cells show signs of attachment to the HAp film coatings and cellular differentiation after 24 h of incubation.

## 4. Discussion

Generally, HAp is an ideal material for dental restorations, as it is the constituent material of the tooth substance [19,28]. It is extremely difficult to develop an artificial HAp enamel for the teeth owing to the difference in roughness. An advanced HAp treatment method uses powder jet deposition, which forms an HAp film by causing HAp particles to collide with the tooth surface at high speed and at room temperature/atmospheric pressure [7]. Additionally, an ultra-thin aperture sheet bonding method has been proposed in which an HAp sheet with a thickness of 1–10 µm is attached to the tooth surface and integrated with the tooth substance; however, this method has not been applied practically [29].

The Er:YAG laser can markedly decrease the unpleasant sounds and vibrations during dental treatment [30]. The wavelength of the Er:YAG laser is as long as 2.94 μm in the region of the major absorption peak for water. Through the micro-explosive reaction of the OOH-Stem of the HAp, the hydration shell inside the crystal is instantly vaporized and thus is the most suitable for hard tissue ablation treatments [31]. In dentistry, the Er:YAG laser is used to cut dentin and remove calculus deposits. The Er:YAG laser is applied to the bulk target of HAp, and particles are scattered from the target, resulting in the formation of an HAp film coating, which is formed by laser deposition on the tooth.

In preliminary experiments, calcium hydrogen phosphate dihydrate, α-TCP, β-TCP, and diphosphoric acid were used as targets for Er:YAG-PLD, but only the α-TCP coating was eventually converted into HAp. Conversely, an HAp film coating from HAp targets cannot achieve the ideal adhesive strength required for enamel and dentin.

Hydrolysis of α-TCP is a dissolution and precipitation process [32,33], and the higher specific surface produced by the reduction of the particle size of the powder strongly accelerates the hydrolysis of α-TCP into calcium-deficient hydroxyapatite [34,35,36]. In the present study, this mechanism was used to pulverize the α-TCP particles (at a size of 1–10 μm) into microparticles (mostly 0.2 μm). The Er:YAG laser irradiation expedited the hydrolysis of the a-TCP into HAp within 48 h.

The roughness values tended to be extremely small, at less than 30 nm for all specimens, and the roughness of HAp film coatings was larger than that of the bovine teeth. Considering the SEM image, we assume that the HAp layer remained after the brushing test. It has been reported that the thickness of softened enamel removed by toothbrushing varies between 254 and 323 nm, depending on the acid used [37]. We speculate that the decrease in roughness is due to the toothbrush grinding the surface of the teeth, smoothing a small number of protrusions. Using the hardness test results, we infer that the HAp film coating was firmly attached to the enamel surfaces, had sufficient wear resistance for daily tooth brushing, and can last for a long time.

Micro-Vickers hardness testing showed that the hardness of the bovine enamel reduced remarkably (from 329 to 164 HV) after etching with 35% phosphoric acid. When the α-TCP sheet was formed on the specimen, 89.4% of the hardness was regained from the initial enamel. A hardness of 91.8% was regained when the crystal turned into HAp. After the Er:YAG laser irradiation, the α-TCP microparticles were expected to occupy the space between the decalcified enamel rods, flatten the surface degradation, and regain the microhardness. However, the HAp film coating formed following Er:YAG-PLD cannot replicate the complicated hierarchical structure of natural enamel. Therefore, further investigations are required to determine whether the Er:YAG-PLD technique can be applied to dental restorations.

To simulate the hydrolysis of α-TCP under intraoral conditions, the bovine tooth slabs were kept in artificial saliva composed of multiple ions at a temperature of 37 °C after Er:YAG-PLD. Peaks of sodium and magnesium ions have also been detected on the bovine enamel surface [38,39]. However, it is clear that the Ca/P value of bovine enamel and HAp coating is lower than 1.67 that of hydroxyapatite crystals. In addition to calcium ions, EDS detected sodium and magnesium ions in bovine enamel and HAp coating, indicating that a small number of calcium ions were replaced by sodium and magnesium ions during the process of hydrolysis of α-TCP performed with saliva as well as the remineralization of bovine teeth.

Temperature elevations of up to 5.5 °C can harm or even destroy the dentin pulp [40,41,42], and Er:YAG-PLD was performed without water mist in this experiment. The temperature change during the Er:YAG-PLD treatment in vitro increased by 0.1 °C in 5 s and 0.4 °C in 10 s. Furthermore, the C400F chip of Er:YAG laser was straightened (retrofit by J. MORITA MFG. CORP) and aimed directly at the α-TCP target, reducing the possibility of falsely targeting healthy tissue. Under the conditions of this study, there were no apparent adverse pulpal effects with the Er:YAG-PLD treatment.

A previous study examined the cell attachment of human mesenchymal stem cells on HAp film coatings deposited by Krypton fluoride pulsed excimer deposition [43]. In the present study, we only investigated the cell attachment of HPdLF cells on an HAp film coating deposited by Er:YAG-PLD. The presence of surface calcium has been reported to play an important role in the adsorption of proteins to specimens through a mechanism involving calcium bridges [44]. Further in vitro studies investigating cell proliferation, cell differentiation, and toxicity assessment are warranted, as the Er:YAG-PLD is designed for clinical application.

In a previous study, the adhesive strength between the HAp film coating and dentin was evaluated by the tensile loading test and was found to be above 3.8 MPa, sometimes even stronger than the adhesive (Araldite) [45]. It was also reported that the bonding strength between the Hap-coated titanium and bone tissues was approximately 3.5 MPa. These values are similar to those of the adhesive strength between the HAp film coating and enamel in the present study. The adhesive strength of the HAp film coating to enamel indicates that the HAp film coating would not be easy to remove in daily life.

## 5. Conclusions

In the present study, a chairside Er:YAG-PLD device was manufactured to form an HAp film coating directly on demineralized bovine enamel under atmospheric pressure and at room temperature, without adverse pulpal effects. Furthermore, specific Er:YAG-PLD conditions were established. We utilized the ablation phenomenon of the Er:YAG laser by irradiating the bulk of α-TCP into microparticles, which were plugged into the interspace of the bovine enamel prisms to recover the loss of hardness. Then, the deposited α-TCP layer was hydrolyzed by artificial saliva to create an HAp film coating. HAp film coatings with a relatively high degree of crystallinity were firmly attached to the bovine enamel surfaces after the brushing tests.

Therefore, we believe that this Er:YAG-PLD technique has the potential for development as a promising enamel repair strategy for dental applications in the future.

## Figures and Tables

**Table 1:** Mean values and standard deviations of the elemental compositions of bovine enamel and hydroxyapatite film coating. (*n* = 5).

Specimen	Element	C K	O K	Na K	Mg K	P K	Cl K	Ca k	Totals
Bovine enamel	Weight %	4.97 ± 0.43	47.18 ± 0.42	1.22 ± 0.24	0.26 ± 0.02	17.14 ± 0.15	0.20 ± 0.01	29.03 ± 0.63	100
	Atomic %	8.79 ± 0.71	62.61 ± 0.27	1.13 ± 0.22	0.23 ± 0.02	11.75 ± 0.19	0.12 ± 0.01	15.38 ± 0.44	100
HAp coating	Weight %	5.60 ± 1.49	41.66 ± 1.06	1.41 ± 0.63	0.28 ± 0.02	17.79 ± 0.32	-	33.25 ± 1.18	100
	Atomic %	10.23 ± 2.58	57.28 ± 1.60	1.35 ± 0.60	0.26 ± 0.02	12.63 ± 0.41	-	18.25 ± 0.87	100

**Table 2:** Calcium-to-phosphorous (Ca/P) ratio of bovine enamel and hydroxyapatite film coating. (*n* = 5).

Specimens	Ca/P
Bovine enamel	1.31 ± 0.02
HAp coating	1.45 ± 0.05

## Data Availability

Not applicable.
